# Dihydro-Resveratrol Attenuates Oxidative Stress, Adipogenesis and Insulin Resistance in In Vitro Models and High-Fat Diet-Induced Mouse Model via AMPK Activation

**DOI:** 10.3390/nu15133006

**Published:** 2023-06-30

**Authors:** Chu-Shing Lam, Yi-Xuan Xia, Bai-Sen Chen, Yin-Xiao Du, Kang-Lun Liu, Hong-Jie Zhang

**Affiliations:** Teaching and Research Division, School of Chinese Medicine, Hong Kong Baptist University, Kowloon, Hong Kong, China; 13207504@life.hkbu.edu.hk (C.-S.L.); xiayixuan@hkbu.edu.hk (Y.-X.X.); 22483136@life.hkbu.edu.hk (B.-S.C.); 17482038@life.hkbu.edu.hk (Y.-X.D.); liukanglun@hkbu.edu.hk (K.-L.L.)

**Keywords:** dihydro-resveratrol, natural product, obesity, diabetes, oxidative stress, adipogenesis, insulin resistance, high-fat diet

## Abstract

Management of obesity has become a prevalent strategy for preventing the diseases closely integrated with excess body weight such as diabetes over the last half century. Searching for therapeutic agents acting on oxidative stress, adipogenesis and insulin resistance is considered as an efficient approach to control obesity-related diseases. The present study was designed to examine the in vitro and in vivo effects of dihydro-resveratrol (DR2), a naturally occurring compound from *Dendrobium* medicinal plants, on oxidative stress aggravation, adipogenesis, lipogenesis and insulin sensitivity. We utilized an in vitro 3T3-L1 adipocyte differentiation model to show that DR2 could reduce pre-adipocyte maturation by activation of AMPK/SIRT1 signaling proteins to inhibit p38MAPK proteins. With the use of in vitro oxidative-stress-induced hepatocytes and myoblasts models, DR2 was also shown to be able to reduce oxidative stress aggravation through mediation of Nrf2-related antioxidative cascade, reduce intracellular lipid accumulation through phosphorylation of ACC protein, reduce lipid peroxidation in hepatocytes and promote insulin sensitivity via activation of AKT protein in the insulin-resistant HepG2 cells and C2C12 cells. The effects of DR2 on adipogenesis, lipid accumulation, insulin resistance and blood glucose clearance were further demonstrated in the high-fat diet-induced obesity mouse model. Our in vitro and in vivo studies determined that DR2 could contain therapeutic potential for the treatment of obesity and type 2 diabetes.

## 1. Introduction

Reactive oxygen species (ROS) are generated as byproducts normally during cell metabolism, which is essential for some physiological roles. However, uncontrolled and overwhelmed production of ROS under certain pathological conditions induces cell oxidative stress, which could lead to damage of cells [1]. Obesity-induced oxidative stress [2] could cause impairment of glucose uptake in muscle [3], progression of chronic liver diseases [4] and reduced insulin secretions from the pancreas [5], and obesity is the consequence of excess calories storage in the body with the energy generated through the consumption of food exceeding its utilization [6]. The excess energy produced during the imbalance of energy production and consumption is then converted to triglycerides, resulting in adipocyte accumulation and weight gain [7,8]. Early stages of obesity are indicated by the enlargement of adipocytes (hypertrophy), while later stages of obesity are characterized by the rise of adipocytes proliferation (hyperplasia) [9,10]. Adipocyte hypertrophy is contributed by uncontrolled differentiation and maturation of preadipocytes [11] which involves CCAAT/enhancer-binding proteins (C/EBPs) and peroxisome proliferator-activated receptor–γ (PPARγ), leading to the rise of lipogenesis-related genes including acetyl CoA carboxylase (ACC) and fatty acid synthase (FASN) [12]. During hypertrophy and hyperplasia, abnormal secretions of hormones and cytokines from the adipose tissue could lead to pathologies such as type 2 diabetes [13]. Hyperglycemia and insulin resistance in tissues such as muscle and liver are the two features of the metabolic syndrome, Type 2 diabetes mellitus (T2DM) [14].

AMPK signaling is suggested to be a druggable and potential therapeutic target against various diseases, as activation of AMPK cascades prevents inflammatory cytokines production to reduce inflammation [15]. Phosphorylation at threonine 172 (Thr172) of the AMPK α subunit causes the AMPK signaling activation [16]. Apart from reducing inflammation, AMPK is also responsible for the regulation of adipogenesis in adipose tissues [12], lipogenesis prevention and control of p38 phosphorylation, as well as inhibition of MAPK signaling in liver diseases [17]. Recent studies also suggested that activation of AMPK could reduce insulin resistance by promoting heme oxygenase-1 (HO-1) levels [18]. AMPK proteins have thus been suggested as one of the protective mechanisms in diabetes treatment [19].

Nuclear factor-erythroid-2-related factor 2 (Nrf2) is a transcription factor that was reported to regulate redox homeostasis with modulation of antioxidant and anti-inflammatory responses [20]. Nrf2 activation is suggested to be a double-edged sword, as Nrf2 encode for antioxidant enzymes and are responsible for ROS cleavage under oxidative stress, while the expression level of Nrf2 is also found to be high in chemo-resistant cancer tissues [21] and contributed to the proliferation of cancer cells and protection against drug-induced oxidative stress [22]. For the treatment of obesity, the roles of Nrf2 on adipogenesis and insulin resistance are still in discussion, but it is suggested that agents that serve as Nrf2 activators could alleviate obesity and related metabolic syndromes [23].

Dihydro-resveratrol (DR2) can be found in the genera of *Dendrobium*, *Dioscorea* and *Bulbophyllum* plants, plants in the *Orchidaceae* family and *Cannabis sativa* L. as an anti-microbial phytoalexin [24,25,26]. Moreover, this stilbene is also found to be a metabolite converted by the gut microbiota from the well-known antioxidant compound resveratrol (RES) through hydrogenation of its double bond [27]. Prior research has indicated the anti-inflammatory and antioxidant effects of DR2 on pancreatitis [28]. However, as far as we know, the effect of DR2 on reducing oxidative stress aggravation in obesity and insulin sensitivity has not been reported previously. Thus, the objective of the present study was to examine the effect of DR2 on oxidative stress aggravation, adipogenesis, lipogenesis and insulin sensitivity through different models to explore the therapeutic potential of DR2 against obesity and type 2 diabetes.

We evaluated the effect of DR2 on preventing adipocyte differentiation in 3T3-L1 cells, and we further demonstrated that DR2 significantly reduced the pre-adipocyte maturation by activation of AMPK/SIRT1 signaling proteins to inhibit p38MAPK proteins, leading to the reduction of C/EBPα, PPARγ and FASN. We also showed that DR2 could reduce oxidative stress aggravation through mediation of Nrf2-related antioxidative cascade, reduce intracellular lipid accumulation through phosphorylation of ACC protein, reduce lipid peroxidation and promote insulin sensitivity via activation of AKT protein in HepG2 cells and C2C12 cells. Our study further showed that DR2 treatment could reduce insulin resistance for faster blood glucose clearance, lipid accumulation and adipocyte differentiation in high-fat diet-induced mouse model. These results clearly demonstrated DR2 as a potential agent for protecting against obesity and type 2 diabetes by activation of AMPK proteins in different models.

## 2. Materials and Methods

### 2.1. Cell Lines and Cell Culture

Mouse preadipocytes 3T3-L1 cells, mouse myoblast C2C12 cells and human hepatocarcinoma HepG2 cells were used for the cytotoxicity evaluation and in vitro experiments. The cell lines were purchased from ATCC and were cultured in Dulbecco’s Modified Eagle Medium (DMEM) (GIBCO, Waltham, MA, USA) with 10% fetal bovine serum (FBS, GIBCO, Waltham, MA, USA), 1% penicillin–streptomycin (PS, GIBCO, Waltham, MA, USA) in a 5% CO_2_, 95% air-humidified atmosphere at 37 °C.

### 2.2. Cell Viability Assay

To determine the cell viability, MTT [3-(4,5-dimethylthiazol-2-yl)-2,5-diphenyl tetrazolium bromide] assay was applied, and cells were plated in 96-well plates with a density of 8000 cells/well and treated with serial concentrations of a compound sample for 48 h prior to MTT assay. Then, 0.5% Dimethyl sulfoxide (DMSO) was used as a vehicle solution. At the time of harvest, the cells were incubated with MTT solution (0.5 mg/mL) at 37 °C for 3 h, and 100% DMSO was used to dissolve the MTT formazan products with some gentle shaking and incubated at r.t. (room temperature) for 0.5 h. Spectrophotometric absorbance of the solubilized samples was determined at a wavelength of 570 nm using a microplate reader (Bio-Rad, Hercules, CA, USA).

### 2.3. Protein Extraction and Western Blot Analysis

3T3-L1/HepG2/C2C12 cells lysed with RIPA Mammalian Protein Extraction Reagent (Thermo Fisher Scientific, Waltham, MA, USA) was supplemented with protease inhibitor cocktail (Thermo Fisher Scientific, Waltham, MA, USA) to extract the total proteins. Next, 20 µg of the lysates were loaded to each lane and separated by 7.5–10% sodium dodecyl sulfate–polyacrylamide gel electrophoresis (SDS–PAGE). After wet electroblotting, proteins were transferred onto polyvinylidene difluoride membranes (PVDF) (Western Bright, Advansta Inc., San Jose, CA, USA). An amount of 5% nonfat milk in Tris-buffered saline containing 0.1% Tween 20 (TBST) was used for the blocking step with 1 h of incubation at r.t. Electro Blots were probed with primary antibodies overnight at 4 °C and then probed with corresponding horseradish peroxidase-conjugated anti-rabbit, anti-goat or anti-mouse secondary antibodies (Bio-Rad, Hercules, CA, USA) and visualized by utilization of an ECL kit (Western Bright, Advansta Inc., San Jose, CA, USA). The primary antibodies used are described in Table 1.

### 2.4. Real-Time Quantitative PCR (qPCR)

Total RNA was extracted using TRIzol reagent (Invitrogen, Waltham, MA, USA) according to the manufacturer’s instructions. A PrimeScript™ RT Master Mix Kit (Takara Bio, Shiga, Japan) was used to turn 1 µg of total RNA into 10 µL of cDNA and SsoFast™ EvaGreen^®^ Supermix (Bio-Rad, Hercules, CA, USA) was used for the qRT-PCR on the Bio-Rad CFX Connect real-time PCR system. Endogenous control *Gapdh* was used for normalization of the expression of gene of interest. The comparative CT (2^−ΔΔCT^) method was used to determine the fold changes, and the primer sequences are listed in Table 2.

### 2.5. Cellular MDA Assay

Cells were lysed after the treatment in a 12-well plate and centrifuged at 12,000 rpm for 15 min to obtain the supernatant. The Lipid Peroxidation (MDA) Assay Kit (Abcam, Cambridge, UK) was used according to the manufacturer’s instructions to measure the cellular MDA content, while a BCA protein assay kit (Thermo Fisher Scientific, Waltham, MA, USA) was used to determine the protein concentration.

### 2.6. Oil Red O Staining

The intracellular lipid aggregation level in cells was observed by Oil /Red O staining. At the time of harvest, cells rinsed with PBS (phosphate-buffered saline)/tissue sections were fixed with 4% paraformaldehyde for 15 min, followed by washing with ddH_2_O (double-distilled water) and stained with Oil Red O working solution (Santa Cruz Biotechnology, Dallas, TX, USA) for 15 min. After washing with ddH_2_O, hematoxylin solution (Sigma-Aldrich, St. Louis, MO, USA) was used to counterstain for 1 min, followed by washing with ddH_2_O three times. The stained cells/slides were observed under light microscope (Leica Microsystems, Wetzlar, Germany).

### 2.7. 3T3-L1 Adipocyte Differentiation

For adipocytes differentiation, 2-day postconfluent cells (day 0) were incubated with the complete medium with supplement of IBMX (3-isobutyl-1-methylxanthine), dexamethasone, insulin and Rosiglitazone (designated hereafter as MDIR). Cells were treated with or without DR2 (20/40/80 μM) or RES (40 μM) for 48 hrs at day 0, together with MDIR differentiation. The medium was changed to postdifferentiation medium (Complete medium with insulin and rosiglitazone) after 48 h (day 2) for 2 days (day 4) and changed with the complete medium every 2 days till day 10 for oil red staining or protein extraction.

### 2.8. H_2_O_2_-Induced Oxidative Stress HepG2 cells

For the H_2_O_2_-inducing oxidative stress challenge, HepG2 cells with a cell density of 2 × 10^5^ cells/well were plated in a 6-well plate and exposed with 100 μM of H_2_O_2_ and treated with or without DR2 (10, 20 or 40 μM) or ascorbic acid (125 μM) for 48 h for Western blot analysis.

### 2.9. Insulin-Resistant HepG2 Cells and C2C12 Cells

To evaluate the effect of DR2 against high-glucose and high-insulin challenge, cell density of 1 × 10^5^ cells/well of HepG2/C2C12 cells were plated in a 12-well plate and exposed with or without 30 mM of glucose, 100 nM of insulin for 48 h together with or without DR2 (10, 20 or 40 μM) or rosiglitazone (rosi) (10 μM), and then they were harvested for the Western blot or MDA assay analysis after stimulation of insulin (100 nM) for 15 min.

### 2.10. Animal Husbandry and Treatment

Male C57BL/6J mice (age, 6–8 weeks, body weight, 20–25 g) were housed in the standard cages in a humidity-controlled environment with a r.t. of 23 ± 2 °C and 12 h light/dark cycle with unlimited food and water. Obesity was induced by feeding on 60 kcal% HFD (Research Diets, Inc., New Brunswick, NJ, USA) for 6 weeks before the DR2 treatment. A standard chow diet (13.5% from fat calories) (Lab Diet, Inc., St. Louis, MO, USA) was given to the control mice. DR2 was dissolved in vehicle solution (0.5% Na-CMC) prior to oral administration. Mice were administrated with vehicle solution, 40 or 80 mg/kg/d DR2 via oral gavage for 5 consecutive days per week for 3 weeks. Ethical approval ((23-1) in DH/HT&A/8/2/6 Pt.7) for all husbandry and experimental procedures had been obtained and was in accordance with the Animals Ordinance, Department of Health, Hong Kong SAR, China.

### 2.11. Intraperitoneal Glucose Tolerance Test (IPGTT)

After fasting for 6 h, the fasting glucose level was checked from venous blood from the tail of the mice. After that, the mice received an intraperitoneal injection of 2 mg/g of glucose solution, and blood glucose levels were determined at the time points of 0, 30, 60, 90 and 120 min to assess the glucose tolerance.

### 2.12. Histological Examination

Upon scarification, liver and inguinal white adipose tissue (iWAT) were immediately removed and frozen with OCT (Optimal cutting temperature) compound. Hematoxylin and eosin (H&E) staining and Oil Red O staining were performed after the sectioning.

### 2.13. Statistical Analysis

The statistical differences were measured with one-way analysis of variance (ANOVA) followed by Tukey’s test as a post hoc test using GraphPad Prism 5. All values are expressed as means ± SEM. A *p* value of <0.05 is accepted as statistically significant.

## 3. Results

### 3.1. Effect of Dihydro-Resveratrol (DR2) on Lipogenesis and Adipogenesis in 3T3-L1 Adipocyte Differentiation Cell Model

#### DR2 Reduces Lipogenesis and Adipogenesis via Modulation of AMPK/SIRT1 and p38 Cell Signaling Pathway in 3T3-L1 Cells

The cytotoxicity of DR2 and RES on 3T3-L1 cells were first examined in terms of mitochondrial metabolism using the MTT cell viability assay. The IC_50_ values of DR2 and RES in 3T3-L1 were measured at 502.5 μM and 162.6 μM, respectively. The cytotoxicity assay result indicated the low cytotoxicity of DR2 to the murine preadipocytes, indicating that DR2 is less harmful to the preadipocytes than RES in vitro.

**Figure 1:**
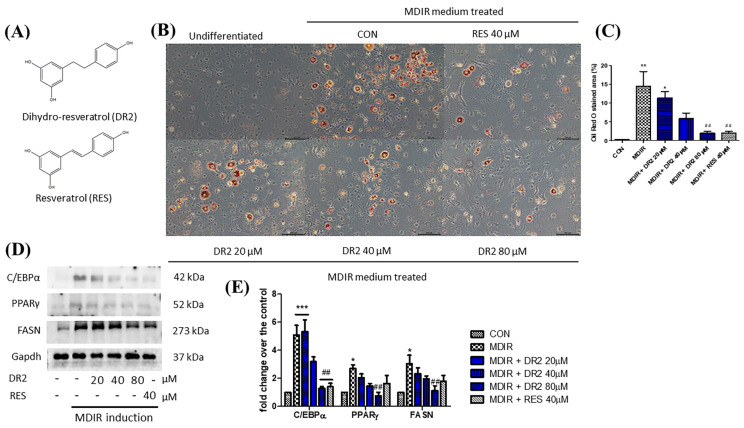
Effect of DR2 on adipocyte differentiation of 3T3–L1 cells. (**A**) Structures of dihydro–resveratrol (DR2) and resveratrol (RES). (**B**) The Oil Red O staining images and (**C**) quantifications of MDIR–stimulated 3T3–L1 cells with or without DR2/RES treatment. Scale bar = 100 μm. (**D**) Western blot results and (**E**) quantifications that indicated that DR2 reduced adipogenesis and lipogenesis markers in 3T3–L1 cells. * *p* < 0.05, ** *p* < 0.01 and *** *p* < 0.001 vs. CON group; ## *p* < 0.01 vs. MDIR group.

**Figure 2:**
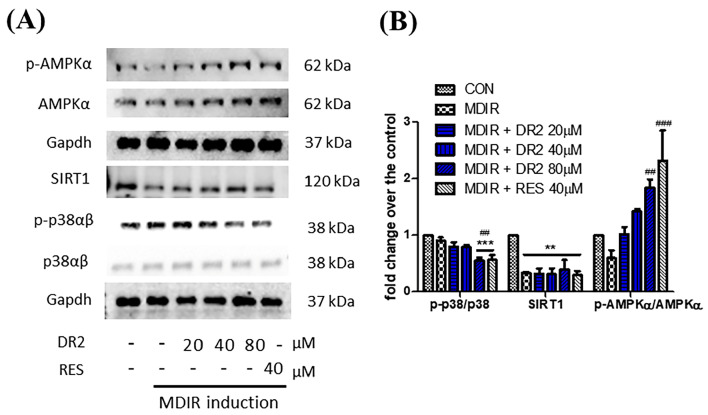
Modulation of cell signaling proteins of DR2 on adipocyte differentiation of 3T3-L1 cells. (**A**) Western blot images and (**B**) quantifications indicated that DR2 reduced adipogenesis and lipogenesis via modulation of AMPK/SIRT1 signaling and phosphorylation of p38αβ in 3T3–L1 cells. ** *p* < 0.01 and *** *p* < 0.001 vs. CON group; ## *p* < 0.01 and ### *p* < 0.001 vs. MDIR group.

Oil Red O staining assay was applied to study the effect of DR2 on lipogenesis and adipogenesis in 3T3-L1 cells. In Figure 1B, we show that treatment with DR2 at 40 and 80 μM for 48 h could reduce the adipocytes differentiation in 3T3-L1 cells, and DR2 at 40 μM possessed a similar effect as RES at 40 μM. Next, we investigated the expressions of proteins related to the lipogenesis and adipogenesis. From the immunoblot images of Figure 1D, we observed that DR2 could downregulate C/EBPα, PPARγ and FASN protein expressions. The results suggested that DR2 could reduce adipogenesis and lipogenesis in adipocytes. We then examined different cell signaling proteins to reveal the mechanisms of DR2. In these assays, we observed that DR2 induced the activations of AMPK and SIRT1 proteins and it could significantly reduce the phosphorylation of p38αβ at 80 μM. These results suggested that DR2 reduced adipocyte differentiation and lipogenesis in 3T3-L1 cells through the phosphorylation of AMPK proteins (Figure 2).

### 3.2. In Vitro Effect of Dihydro-Resveratrol (DR2) on Oxidative Stress HepG2 Cell Model

#### 3.2.1. DR2 Mediates Nrf2-Related Antioxidative Cascade by Activation of AMPK/SIRT1 Signaling Proteins in HepG2 Cells

We first examined the cytotoxicities of DR2 and H_2_O_2_ on HepG2 cells by the MTT cell viability assay. The IC_50_ values of DR2 and H_2_O_2_ in HepG2 cells were measured at 558.7 μM and 541 μM, respectively.

**Figure 3:**
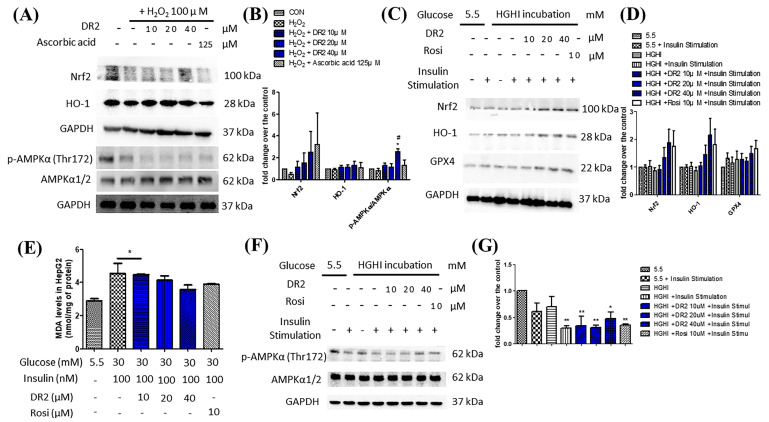
Effects of DR2 on oxidative stress markers in HepG2 cells. Western blot results and quantifications that indicated that DR2 reduced oxidative stress markers via phosphorylation of AMPKα in (**A**,**B**) H_2_O_2_–induced HepG2 cells and (**C**–**G**) high–glucose high–insulin–exposed HepG2 cells. (**E**) MDA levels after DR2 treatment in high glucose high insulin exposed HepG2 cells. * *p* < 0.05 and ** *p* < 0.01 vs. CON group; # *p* < 0.05 vs. H_2_O_2_ group.

To examine the effects of DR2 on Nrf2 and its related antioxidative proteins, we treated the HepG2 cells with 100 μM of H_2_O_2_, followed by various concentrations of DR2 (10, 20 or 40 μM) or 125 μM of ascorbic acid for 48 h for Western blot analysis. From Figure 3A, we observed that the Nrf2 expression level was reduced for the H_2_O_2_-treated sample when comparing to the untreated control, while such reduction of Nrf2 could be rescued by dose-dependent DR2 treatments. HO-1 is one of the major antioxidant enzymes mediated by Nrf2, and we observed that DR2 enhanced the protein expression of HO-1 in the H_2_O_2_-challenged HepG2 cells (Figure 3A). These results clearly showed that the antioxidant effects of DR2 are mediated though Nrf2.

We also studied the effects of DR2 on the regulation of AMPK proteins. From Figure 3A, we observed that DR2 treatment could lead to dose-dependent upregulation of phosphorylated AMPKα (Thr172) and SIRT1. The results suggested that the effect of DR2 on activation of antioxidants is associated with activation of AMPK/SIRT1 cell signaling.

#### 3.2.2. DR2 Treatment Upregulates the Antioxidant Protein Levels of High-Glucose High-Insulin (HGHI)-Exposed HepG2 Cells to Reduce Oxidative Stress Aggravation via Activation of AMPK Protein

It is suggested that high glucose levels will increase ROS in cells and cause oxidative stress to the cells [29], leading to lipid peroxidation. Therefore, we examined the lipid peroxidation levels in HGHI-exposed HepG2 cells by using MDA assays. We observed that the MDA levels of HGHI-exposed cells were significantly upregulated when compared with HepG2 cells in the normal condition, while the DR2 treatment (10, 20 and 40 μM) could dose-dependently reduce the MDA levels, suggesting that DR2 treatment could reduce the ROS levels as well as lipid peroxidation in HGHI-exposed HepG2 cells (Figure 3E). We examined the expression levels of AMPK protein and the antioxidant proteins Nrf2, HO-1 and GPX4 in the HepG2 cells to reveal the reducing effect of DR2 on oxidative stress aggravation. We found that treatment of DR2 could upregulate Nrf2, HO-1, GPX4 and p-AMPK (Thr172) protein expressions dose-dependently. Our results demonstrated that DR2 could reduce the oxidative stress and lipid peroxidation by activating Nrf2, HO-1 and GPX4 proteins via phosphorylations of AMPK protein in HepG2 cells (Figure 3C,F).

#### 3.2.3. DR2 Treatment Reduces Intracellular Lipid Aggregation in Oleic Acid-Treated HepG2 Cells through Activation of AMPK Proteins

**Figure 4:**
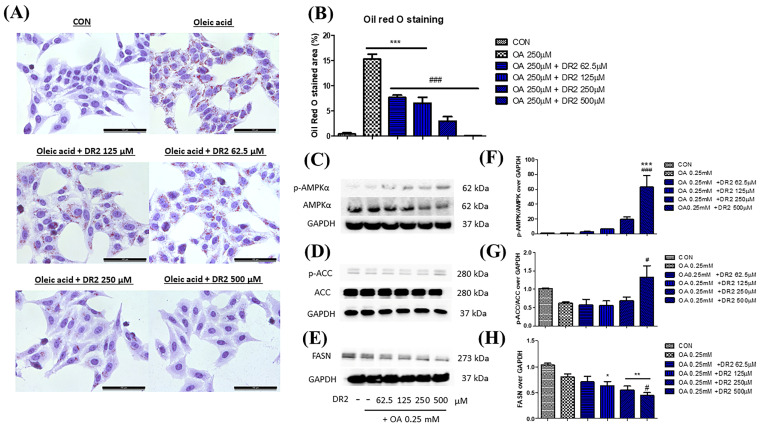
Effect of DR2 treatment on oleic acid–stimulated HepG2 cells. (**A**) The Oil Red O staining images and (**B**) quantifications of oleic acid–stimulated HepG2 cells with or without DR2 treatments (62.5 µM to 500 µM). Scale bar = 100 μm. (**C**–**E**) Western blot results and (**F**–**H**) quantifications that indicated that DR2 is an in vitro ACC inhibitor via modulation of ACC/AMPK signaling in HepG2 cells. * *p* < 0.05, ** *p* < 0.01 and *** *p* < 0.01 vs. CON group; # *p* < 0.05 and ### *p* < 0.001 vs. OA group.

Oil Red O staining was used to demonstrate the effect of DR2 on intracellular lipid accumulation. From Figure 4A, oleic acid (OA) at 250 μM could significantly induce lipid deposition in HepG2 cells, as the lipid droplets (stained in red) were greatly increased when compared with the control, and such elevation of lipid droplets accumulations could be reduced by DR2 treatments starting at 62.5 μM. Next, we investigated the expressions of proteins from the AMPK/ACC cell-signaling pathway. From the immunoblot images, we observed that DR2 could increase the p-AMPKα (Figure 4C) and p-ACC (Figure 4D) protein expressions, leading to the inhibition of its downstream target, Fatty Acid Synthase (FASN) (Figure 4E). The results suggested that DR2 could reduce intracellular lipid accumulation by activation of the AMPK/ACC signaling pathway.

### 3.3. In Vitro Effect of Dihydro-Resveratrol (DR2) on Insulin Sensitivity in Insulin-Resistant Cell Model

#### DR2 Treatment Reverses the Reduced AKT Levels of High-Glucose High-Insulin (HGHI)-Treated HepG2 Cells to Ameliorate the Insulin Resistance

**Figure 5:**
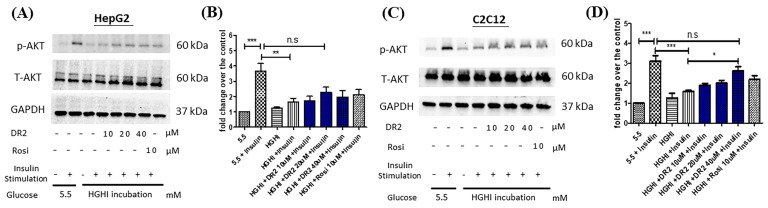
Effect of DR2 on insulin sensitivity of HGHI–exposed cell models. Western blot results that indicated that DR2 increased insulin sensitivity of insulin-resistant (**A**,**B**) HepG2 cells and (**C**,**D**) C2C12 cells via upregulations of phosphorylated AKT protein expression levels**.** Statistical significance considered as * *p* < 0.05, ** *p* < 0.01, *** *p* < 0.001 and n.s as no significance.

To examine the effect of DR2 on ameliorating the insulin resistance of cell models, insulin-resistant HepG2 cells were first established by incubation with high-glucose (30 mM), high-insulin (100 nM) or normal condition (5.5 mM of glucose) for 48 h before protein extraction. Before harvesting the proteins, the insulin responses of HepG2 cells were triggered by stimulation of 100 nM of insulin for 15 min. From Figure 5A, we observed the significant upregulations of p-AKT (ser473) levels for the normal HepG2 cells with insulin stimulation, while the upregulations of p-AKT levels of high-glucose high-insulin (HGHI)-exposed HepG2 cells after insulin stimulation were significantly lower than those of normal HepG2 cells, suggesting that the insulin-resistant model was successfully established in HepG2 cells. We also observed that treatment of DR2 (20 or 40 μM) could improve the insulin sensitivity of HGHI-exposed HepG2 cells by increasing the phosphorylation of AKT levels upon insulin stimulation, and such effects of DR2 at 20 or 40 μM were compatible to the effects of the positive control, rosiglitazone (rosi) at 10 μM. Next, we also induced the insulin resistance by a similar approach in the murine muscle cell line C2C12. From Figure 5C, we observed significant upregulations of p-AKT (ser473) levels for the normal C2C12 cells with insulin stimulation, while the upregulations of p-AKT levels of high-glucose high-insulin (HGHI)-exposed C2C12 cells after insulin stimulation were significantly lower than those of normal cells, suggesting that the insulin-resistant model was successfully established in in C2C12 cells. Our results showed that DR2 treatments could dose-dependently increase the insulin sensitivity in the insulin-resistant C2C12 cells as DR2 at 40 μM could rescue the phosphorylated AKT levels significantly. These data suggested that DR2 could enhance AKT activation after insulin stimulation and promote insulin sensitivity in the insulin-resistant cells.

### 3.4. Effect of Dihydro-Resveratrol (DR2) on High-Fat Diet (HFD)-Induced Obesity Mice

C57BL/6J mice were fed with a high-fat diet (HFD) for 6 weeks to establish diet-induced obesity for investigation of the in vivo anti-obesity effect of DR2. Mice that received HFD were then separated into three groups (vehicle and two DR2 treatment groups). Treatment groups were receiving oral administration of DR2 (dissolved in 0.5% CMC-Na) at 40 mg/kg or 80 mg/kg. Mice were treated with a sample daily for 5 consecutive day per week for 3 weeks.

#### 3.4.1. DR2 Treatment Reduces Percentage Weight Gain and Reduces Glucose Intolerance in the Model Mice

**Figure 6:**
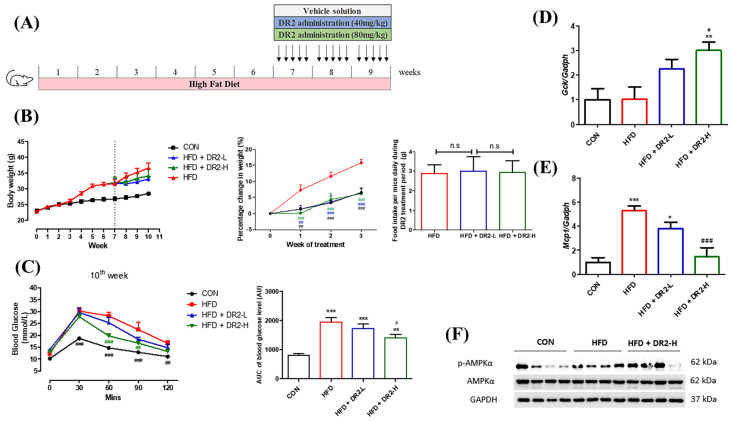
Effects of DR2 treatment on body weights and blood glucose clearance of HFD-treated C57/BL6 mice. (**A**) DR2 treatment period indication in high–fat–diet–fed mouse model. (**B**) Body weights of the experimental groups over the periods, percentage change of weights after DR2 or vehicle solution treatment and daily food intake between the high–fat–diet groups during treatment. (**C**) Intraperitoneal glucose tolerance test after 3 weeks of DR2 treatment. (**D**) qPCR analysis of liver *Gck* transcripts after 3 weeks of DR2 treatments. (**E**) qPCR analysis of iWAT *Mcp1* transcripts after 3 weeks of DR2 treatments. N = 6–8 (**F**) Western blot result of hepatic p–AMPK and AMPK protein expression level after 3 weeks of DR2 treatments. N = 4 * *p* < 0.05, ** *p* < 0.01 and *** *p* < 0.001 vs. CON group; # *p* < 0.05, ## *p* < 0.01 and ### *p* < 0.001 vs. HFD group; n.s as no significance.

C57BL/6J mice were found to demonstrate significant body weight gain after feeding on a high-fat diet (HFD) for 6 weeks. The mice were grouped evenly to receive DR2 treatments or vehicle solutions with free access to HFD for an additional 3 weeks. The mice that received DR2 treatments showed significant reduced percentage weight gain compared to the HFD diet group without affecting the daily food consumption per mice (g), indicating that DR2 possessed weight-control ability against diet-induced weight gain, and such an effect was not due to a reduction of food intake (Figure 6B). The blood glucose level of mice with a high dosage of DR2 treatment dropped significantly at the time points of 60 and 90 min when compared to HFD mice during IPGTT (Figure 6C). These results suggested that DR2 treatments could reduce body weight increment, improve glucose tolerance, and promote faster blood glucose clearance in the obesity mice.

To investigate how DR2 treatment promotes faster blood glucose clearance, quantitative real-time qPCR (RT-qPCR) was used to determine different gene expressions related to glucose homeostasis and insulin resistance. A high dosage of DR2 treatment enhanced the *Gck* transcription significantly in the liver of the mice (Figure 6D), and it also significantly reversed the upregulated *Mcp1* gene transcription in the inguinal white adipose tissue (iWAT) of model mice (Figure 6E). These results suggested that DR2 treatment could target multiple tissues against obesity-induced insulin resistance, including liver *Gck* and white adipose tissue *Mcp1.* We also tested the protein expression levels of p-AMPK and AMPK in the liver tissues, and the result indicated that a high dosage of DR2 treatment could induce AMPK activation (Figure 6F).

#### 3.4.2. DR2 Treatment Reduces Adipogenesis and Lipid Aggregation in the Model Mice

**Figure 7:**
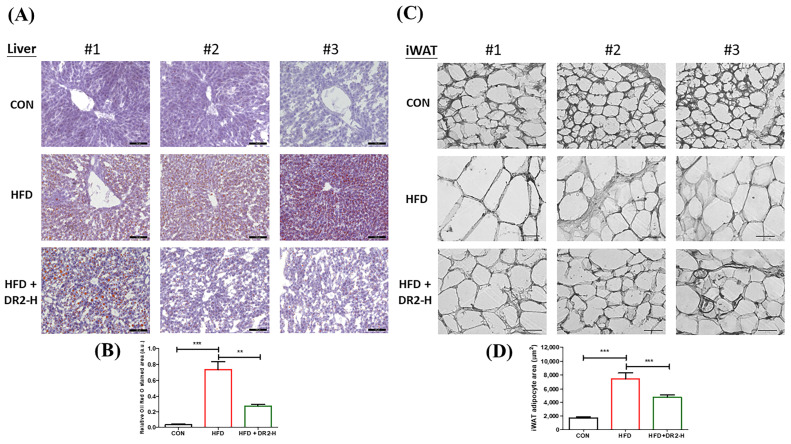
Effects of DR2 treatment on lipid aggregation and adipogenesis of HFD-treated C57/BL6 mice. (**A**) Hepatic Oil Red O staining images with (**B**) quantifications (200×). Scale bar = 100 μm. (**C**) H&E staining images and (**D**) quantification of iWAT tissues (400×). Scale bar = 50 μm. N = 6–8. Statistical significance considered as ** *p* < 0.01 and *** *p* < 0.001.

We employed tissue sectioning of liver tissues and inguinal white adipose tissues (iWAT) to visualize the lipid accumulation and adipocyte size to demonstrate the effects of DR2 on lipid aggregation and adipogenesis. We examined the effect of DR2 on hepatic lipid aggregation by Oil Red O staining. The visualized and quantitative data showed that the high-dosage DR2 treatment significantly reduced the enhanced hepatic lipid deposition of the high-fat-diet-fed mice (Figure 7A). From the H&E staining, a significant rise in the adipocyte size of the HFD group was observed while high-dosage treatment of DR2 could significantly reduce the adipocyte enlargement (Figure 7C). All these results were aligned with the results obtained through the in vitro cell models and demonstrated the effectiveness of DR2 administration in preventing lipid accumulation and adipocyte hypertrophy.

## 4. Discussion

Pharmacological modulation of AMPK protein could be one of the therapeutic approaches to treat different diseases [16], as AMPK activation is essential in oxidative stress regulation of the cells, which is suggested to related to more than 100 pathologies [30] including diabetes, and pancreatic and liver disorders. Studies have suggested that AMPK mediates antioxidative events by activation of Nrf2 [31], leading to the translocation of Nrf2 into the nucleus. Nrf2 takes part in the cellular antioxidant defense system by inducing other cytoprotective proteins like GPX4 and HO-1 [32]. HO-1 is the rate-limiting enzyme that is responsible for the catalyzation of heme degradation to generate potent antioxidants [32], while GPX4 is regarded as the key downstream marker for the system Xc–/GSH/GPX4 axis, as it is the sole glutathione peroxidase that is known to be responsible for intracellular lipid peroxide reduction now [33]. Not only can it prevent oxidative stress aggravation, but AMPK activation is also suggested to be able to inhibit adipogenesis [12], and lipogenesis [17], and promote insulin sensitivity [14]. Thus, the effects of DR2 on activation of AMPK proteins in different cell models were examined to observe its anti-obesity and antioxidant effects to explore the therapeutic potential of DR2 on the treatment of obesity and type 2 diabetes in this study.

For the in vitro adipogenic model, 3T3-L1 preadipocytes is one of the most used models to explore the therapeutic potential for a compound as an anti-obesity agent [34]. Prior research studies have demonstrated that the preadipocytes could turn into mature white adipocytes in response to stimulation of IBMX, dexamethasone and insulin [35,36], or together with rosiglitazone [37] to induce peroxisome proliferator-activated receptor γ (PPAR-γ) and CCAAT/enhancer-binding protein α (C/EBPα) protein expressions as well as adipocyte differentiation [34] and lipid droplet accumulations. Increases of PPAR-γ and C/EBPα protein expressions also boost lipogenesis by enhancing lipogenic markers such as ACC and FASN [34]. Some studies suggested that adipocyte SIRT1 also takes part in glucose homeostasis and insulin sensitivity [38], while phosphorylation of p38MAPK proteins is also linked to adipocyte differentiation [34]. Our results demonstrated the promoted adipocyte differentiation of 3T3-L1 cells by MDIR combinations to upregulate PPAR-γ, C/EBPα and FASN protein expressions levels through Western blotting, and Oil Red O staining was conducted to show the lipid droplet accumulations level in adipocytes. We demonstrated that DR2 treatment reduced adipogenesis, lipogenesis and lipid droplets by reducing PPAR-γ, C/EBPα and FASN expressions via activations of AMPK/SIRT1 proteins and inhibition of phosphorylation of p38 proteins, which revealed that DR2 treatment could inhibit obesity progression by regulations of adipogenesis and lipogenesis in adipocytes.

For the in vitro hepatic cell models, HepG2 cells is commonly used for evaluating the activities of molecules in reducing oxidative stress aggravation, lipogenesis, and insulin resistance in NAFLD and type 2 diabetes. Hydrogen peroxide (H_2_O_2_) has been widely used as the source of inducing oxidative injury to different cell models, as it can be easily transformed into a destructive hydroxyl radical. The overproduction of oxidative stress as well as ROS cause the depletion of antioxidants and ultimately lead to the breakdown and failure of the cell antioxidant defense system [39]. Nrf2 plays a crucial role in cellular antioxidative defense mechanism [40]. Upon oxidative stress, Nrf2 will be transported into the nucleus and encodes heme oxygenase 1 (HO-1), one of the antioxidant enzymes [41]. In this study, we successfully showed that the treatment of DR2 at the concentrations of 10, 20 or 40 μM could cause dose-dependent upregulation of both Nrf2 and HO-1 protein expressions in HepG2 cells upon the H_2_O_2_ challenge. We believed that the antioxidant effect of DR2 was due to AMPK activations, as our results also demonstrated that DR2 could dose-dependently promote the phosphorylation of AMPK proteins.

Oxidative stress is also a key mechanism linked to insulin resistance [42]. Since DR2 has shown promising AMPK upregulation and antioxidants effects in HepG2 cells, we also tested the activity of DR2 on promoting the responsiveness to insulin stimulation in insulin resistant cell models. Insulin resistance is the major cause of obesity and type 2 diabetes [43]. Different in vitro insulin resistant cell models such as insulin-resistant HepG2 and insulin-resistant C2C12 cell models have been extensively studied to reveal the underlying cellular and molecular mechanisms [29].

For our in vitro insulin-resistant liver cell model, HepG2 cells were grown in a high-glucose and high-insulin environment as high-glucose concentrations trigger insulin resistance by Akt inhibition, while chronic insulin exposure decreases the activity of insulin receptors which contribute to the insulin resistance for the cell model [29]. In our study, we demonstrated that DR2 could increase the insulin sensitivity as the compound at the concentrations of 10, 20 or 40 μM could dose-dependently rescue the Akt phosphorylation upon the insulin stimulation in the insulin-resistant HepG2 cells. We further investigated the antioxidant protein marker expression levels including Nrf2, HO-1 and GPX4 in the HepG2 cells. Our results showed that DR2 treatment could upregulate theses antioxidant markers and their key signaling marker, phosphorylated AMPK. Apart from the antioxidant markers, we also tested the in vitro malondialdehyde (MDA) levels in the insulin-resistant cells. MDA is a common biomarker for lipid peroxidation and oxidative stress [44]. Our results showed that DR2 at the concentrations of 10, 20 or 40 μM could dose-dependently reduce the MDA production in HepG2 cells. These results were aligned with our H_2_O_2-_induced HepG2 cells model and suggested that DR2 could reduce the oxidative stress and promote insulin sensitivity in liver cells.

For our in vitro insulin-resistant muscle cell model, C2C12 myotube cells were used and incubated in a high-glucose and high-insulin concentrations as responses to insulin were also found to be high in the normal mouse C2C12 cells [45]. Our result showed that deteriorated Akt phosphorylation in the C2C12 cells upon insulin stimulation after the exposure of high-glucose and high-insulin condition and such deteriorated phosphorylation of Akt proteins could be significantly rescued by the treatment of DR2 at the concentration of 40 μM. These results provided strong evidence that DR2 could promote the insulin sensitivity in the insulin-resistant muscle cell model.

Obesity, glucose intolerance, and insulin resistance could be induced by chronic high-fat diet [46]. We first fed the mice with 6 weeks of high-fat diet followed by administration of DR2 for 3 more weeks with unlimited access of high-fat diet. Our results demonstrated that DR2 (80 mg/kg) treatments could ameliorate obesity by reducing the rate of body weights increment, size of adipocytes in iWAT and lipid accumulations in liver. Our data showed that the reduction in body weight increment for high-dosage DR2 treatment was not due to the reduced food intake as there is no significant difference between the daily food intake of each group. We believed that the increased energy expenditure contributed to the reduced body weight increment of treatment group as our result indicated the AMPK activation after 3 weeks of DR2-H treatment in the hepatic tissues while further validation and detailed studies on the effect of DR2 on energy expenditure should be done in the future. Our result showed that high dosage of DR2 treatments could lead to the hepatic AMPK activation and reduce the lipid content in the liver. Such results were aligned with the study result using our in vitro HepG2 model. Our in vivo model also showed that the high dosage of DR2 treatments could improve the glucose tolerance and promote faster blood glucose disposal of the high-fat diet fed mice. Overexpression of Monocyte Chemoattractant Protein-1 (MCP-1) in adipose tissue was found to be correlated to insulin resistance and macrophage infiltration [47] while insulin-resistant adipocytes promote *Mcp1* transcription in adipocytes resulting in enhanced production of *Mcp1* and inflammation in adipose tissue [48]. Our results showed that DR2 treatment reversed the upregulated *Mcp1* gene transcript level in iWAT of the HFD fed mice. Glucokinase (GCK) has been suggested to affect blood glucose levels while hyperglycemia can be mimicked by GCK activators [49]. In our mouse model study, DR2 treatment could significantly promote the hepatic *Gck* transcript levels. All these results suggested that DR2 could reduce insulin resistance and promote faster blood glucose clearance by increasing the hepatic uptake of glucose against diet induced obesity.

The in vivo data shown in the mouse model were aligned with the results of DR2 in different in vitro models, which suggested DR2 has promising effects in attenuating oxidative stress, adipogenesis and insulin resistance.

## 5. Conclusions

To conclude, we demonstrated that DR2 (1) reduced adipogenesis via activations of AMPK/SIRT1 proteins and inhibition of phosphorylation of p38 proteins in adipocytes, (2) reduced oxidative stress and lipid accumulations through AMPK activation in hepatocytes, (3) promoted insulin sensitivity in insulin-resistant hepatocytes and skeletal muscle cells. Such effects of DR2 in adipogenesis, lipid accumulation, and blood glucose clearance were further validated in the high-fat diet fed mouse model. Our in vitro and in vivo studies have thus indicated the medicinal potential of DR2, and further research is thus warranted to advance DR2 as a therapeutic agent against obesity and its related diabetes.

## Figures and Tables

**Table 1:** Primary antibodies for Western blotting.

Protein Name	Dilutions	Product Number, Lot Number and Manufacturer
C/EBPα	1:1000	8178, 3, Cell Signaling Technology, Danvers, MA, USA
PPARγ	1:1000	2443, 4, Cell Signaling Technology, Danvers, MA, USA
FASN	1:1000	3180, 7, Cell Signaling Technology, Danvers, MA, USA
Phosphor ACC (Ser79)	1:1000	3661, 10, Cell Signaling Technology, Danvers, MA, USA
ACCA	1:1000	269273, GR3340965-1, Abcam, Cambridge, UK
AMPKα1/AMPKα2	1:1000	A17290, 5500004207, Abclonal, Woburn, MA, USA
Phosphor AMPKα (Thr172)	1:1000	2535, 21, Cell Signaling Technology, Danvers, MA, USA
Total p38∝β	1:500	sc-7972, B2117, Santa Cruz Biotechnology, Dallas, TX, USA
Phosphor p38∝β	1:500	sc-166182, E0117, Santa Cruz Biotechnology, Dallas, TX, USA
Sirtuin 1	1:1000	ab189494, GR3250046-9, Abcam, Cambridge, UK
Nrf2	1:1000	ab62352, YI110703CS, Abcam, Cambridge, UK
HO-1	1:1000	43966, 2, Cell Signaling Technology, Danvers, MA, USA
GPX4	1:1000	ab125066, GR3369574-4, Abcam, Cambridge, UK
Total Akt 1/2/3 antibody	1:1000	44-609G, 2049101, Invitrogen, Waltham, MA, USA
Phospho Akt 1/2/3 (Ser 473)	1:1000	4060, 19, Cell Signaling Technology, Danvers, MA, USA
GAPDH	1:3000	AHP-1628, 155201, Bio-rad, Hercules, CA, USA

**Table 2:** Primers used for qRT-PCR.

	Gene	Forward and Reserve Primer (5′→3′)
Mouse	*Gck*	F: CCCTGAGTGGCTTACAGTTCR: ACTGATGTGAGTGTTGAAGC
	*Mcp1*	F: AGCCAACTCTCACTGAAGCCR: AGCTTGGTGACAAAAACTACAGC
	*Gapdh*	F: CATCACTGCCACCCAGAAGACTGR: ATGCCAGTGAGCTTCCCGTTCAG

## Data Availability

The data presented in this study are available on request from the corresponding authors.
